# Identifying Child Anxiety Through Schools-identification to intervention (iCATS-i2i): protocol for a cluster randomised controlled trial to compare screening, feedback and intervention for child anxiety problems to usual school practice

**DOI:** 10.1186/s13063-022-06773-0

**Published:** 2022-10-22

**Authors:** Tessa Reardon, Obioha C. Ukoumunne, Mara Violato, Susan Ball, Paul Brown, Tamsin Ford, Alastair Gray, Claire Hill, Bec Jasper, Michael Larkin, Ian Macdonald, Fran Morgan, Jack Pollard, Michelle Sancho, Falko F. Sniehotta, Susan H. Spence, Paul Stallard, Jason Stainer, Lucy Taylor, Victoria Williamson, Emily Day, Jennifer Fisk, Iheoma Green, Gemma Halliday, Ciara Hennigan, Samantha Pearcey, Olly Robertson, Cathy Creswell

**Affiliations:** 1 Departments of Experimental Psychology and Psychiatry, University of Oxford, Oxford, UK; 2 NIHR ARC South West Peninsula (PenARC), University of Exeter, Exeter, UK; 3 Health Economics Research Centre, Nuffield Department of Population Health, University of Oxford, Oxford, UK; 4 Bransgore C of E Primary School, Bransgore, UK; 5 University of Cambridge and Cambridge and Peterborough Foundation Trust, Cambridge, UK; 6 School of Psychology & Clinical Language Sciences, University of Reading, Reading, UK; 7 Parents and Carers Together, Suffolk, UK; 8 Life and Health Sciences, Aston University, Birmingham, UK; 9 Charlie Waller Trust, Thatcham, UK; 10 Square Peg, East Sussex, UK; 11 West Berkshire Council, Newbury, UK; 12 NIHR Policy Research Unit Behavioural Science, Newcastle University, Newcastle upon Tyne, UK; 13 School of Applied Psychology and Australian Institute of Suicide Research and Prevention, Griffith University, Brisbane, Australia; 14 Department of Health, University of Bath, Bath, UK; 15 Stanley Primary School, Strathmore Road, London, UK; 16 Oxford NHS Foundation Trust, Oxford, UK; 17 Institute of Psychiatry, Psychology and Neuroscience, King’s College London, London, UK

**Keywords:** Anxiety, Children, Screening, Schools, Identification, Early intervention, Online intervention, Parent-led intervention, Cost-effectiveness

## Abstract

**Background:**

Systematically screening for child anxiety problems, and offering and delivering a brief, evidence-based intervention for children who are identified as likely to benefit would minimise common barriers that families experience in accessing treatment. We have developed a short parent-report child anxiety screening questionnaire, and procedures for administering screening questionnaires, sharing screening outcomes with families, and offering and delivering a brief parent-led online intervention (OSI: Online Support and Intervention for child anxiety) through schools. This trial aims to evaluate clinical and health economic outcomes for (1) children (aged 8–9) who screen positive for anxiety problems at baseline (target population) and (2) the wider population of all children in participating classes (total population) in schools randomly allocated to receive identification-to-intervention procedures and usual school practice (‘screening and intervention’), compared to assessment and usual school practice only (‘usual school practice’).

**Methods:**

The trial design is a parallel-group, superiority cluster randomised controlled trial, with schools (clusters) randomised to ‘screening and intervention’ or ‘usual school practice’ arms in a 1:1 ratio stratified according to the level of deprivation within the school. We will recruit schools and participants in two phases (a pilot phase (Phase 1) and Phase 2), with progression criteria assessed prior to progressing to Phase 2. In total, the trial will recruit 80 primary/junior schools in England, and 398 children (199 per arm) who screen positive for anxiety problems at baseline (target population). In schools allocated to ‘screening and intervention’: (1) parents/carers will complete a brief parent-report child anxiety screening questionnaire (at baseline) and receive feedback on their child’s screening outcomes (after randomisation), (2) classes will receive a lesson on managing fears and worries and staff will be provided with information about the intervention and (3) parents/carers of children who screen positive for anxiety problems (target population) will be offered OSI. OSI will also be available for any other parents/carers of children in participating classes (total population) who request it. We will collect child-, parent- and teacher-report measures for the target population and total population at baseline (before randomisation), 4 months, 12 months and 24 months post-randomisation. The primary outcome will be the proportion of children who screen positive for anxiety problems at baseline (target population) who screen negative for anxiety problems 12 months post-randomisation.

**Discussion:**

This trial will establish if systematic screening for child anxiety problems, sharing screening outcomes with families and delivering a brief parent-led online intervention through schools is effective and cost-effective.

**Trial registration:**

ISRCTN registry ISRCTN76119074. Prospectively registered on 4.1.2022.

**Supplementary Information:**

The online version contains supplementary material available at 10.1186/s13063-022-06773-0.

## Background and rationale

Epidemiological studies report high prevalence rates for anxiety disorders among children and adolescents [1, 2]. The most recent national survey data in England indicates that about one in twenty-five children aged 5 to 10 years have an anxiety disorder [3], and anxiety disorders are among the most common types of disorder experienced by primary-school-aged children [3]. Anxiety disorders during childhood disrupt peer relationships, educational outcomes and family functioning [4–6], and place children at increased risk for ongoing anxiety disorders and other mental health problems [7]. The high prevalence and negative consequences associated with anxiety disorders also create substantial economic costs [8] emphasising the need for effective early identification and intervention.

Cognitive behavioural interventions are effective for children with anxiety disorders [9], but few children with anxiety disorders access evidence-based interventions [10]. Families and GPs [10, 11] report difficulties identifying anxiety problems in children, and in particular differentiating age-appropriate fears and worries from anxiety that causes interference in daily life. Parents report uncertainty about how, when and where to seek professional support, concerns about possible negative consequences of ‘labelling’, and logistical barriers related to attending therapy appointments [12, 13]. Where parents raise concerns about their child’s anxiety with school staff or GPs, they commonly report that their concerns are dismissed, or their child’s difficulties do not meet criteria required to access oversubscribed specialist mental health services. There is growing recognition that schools are ideal settings for delivering evidence-based mental health interventions [14, 15]. Providing support for anxiety problems within school settings would minimise key barriers related to navigating a complex help-seeking process to access services elsewhere. Given the identification barriers associated with anxiety problems, it is essential that school-based interventions for child anxiety problems incorporate a systematic approach to identifying children to target. Equally, it is critical that procedures for offering and delivering support are developed together with families and school staff to maximise acceptability and engagement. Currently, there is no established approach for ‘identification-to-intervention’ for child anxiety problems in primary schools in England.

In underpinning work, we have (1) developed a short questionnaire that is suitable to use for systematic screening of anxiety problems in primary schools [16], (2) worked together with parents/carers, children, school staff and other stakeholders to co-design and refine procedures for administering anxiety screening questionnaires, sharing screening outcomes with families and offering and delivering a brief intervention for children who are identified as likely to benefit [17], and (3) tested the feasibility of evaluating these identification-to-intervention procedures for child anxiety problems in a large randomised controlled trial [18]. Our measure development study identified a 2-item parent-report child anxiety questionnaire (iCATS-2) that is able to identify children with and without anxiety disorders in a community sample with 76% sensitivity and 80% specificity. This measure achieved similar sensitivity and specificity to existing parent-report questionnaires but with the advantage of brevity. Neither child- nor teacher-reported child anxiety questionnaire measures achieved sufficient accuracy for screening purposes (both achieved < 70% sensitivity and specificity), but our co-design work highlighted the importance of involving children and teachers in the process so we will also collect child- and teacher-report questionnaires. As our parent-report screening tool will miss some children who are experiencing anxiety problems (false negatives), support will also be made available on request for any families where the child does not ‘screen positive’ but feel they may benefit. For primary-school-aged children, delivering interventions directly to parents/carers is an effective and cost-effective approach [19, 20] that minimises barriers and concerns related to children attending therapy appointments. The intervention offered in our identification-to-intervention procedures is an online version of an established parent-led treatment for child anxiety disorders (OSI: Online Support and Intervention for child anxiety) [21] that involves parents working through seven online modules, supported by short weekly telephone calls with a Children’s Wellbeing Practitioner (CWP) and a follow-up review a month later. The remote delivery removes barriers related to attending face-to-face appointments, and the digital format provides an opportunity to maximise the accessibility of intervention content by, for example, using videos and animations to demonstrate strategies. In our development work, parents and school staff emphasised the importance of also making some support available to those children whose parents may not participate in the intervention so, in parallel to the targeted support offered directly to parents through OSI, a whole-class lesson on identifying and managing fears and worries, and information for school staff on skills and strategies parents learn through OSI are also provided. Our feasibility study [18] coincided with COVID-19-related restrictions and associated disruption in primary schools which limited the extent to which we could draw firm conclusions related to some feasibility outcomes (e.g. recruitment rates), but the procedures for screening, feedback and intervention delivery were well received by children, parents/carers and school staff in participating classes, and pre-post-intervention questionnaire responses indicated positive effects for families who received OSI [22].

## Objectives

This trial aims to establish whether screening, feedback and intervention for child anxiety problems alongside usual school practice (‘screening and intervention’-intervention arm) bring clinical and health economic benefits compared to assessment and usual school practice alone (‘usual school practice’-control arm). Our target population is children in Year 4 (aged 8–9) who screen positive for anxiety problems at baseline, but we also set out to examine wider potential benefits and evaluate outcomes for all children in participating classes (total population).

Our primary objective is to compare the proportion of children in the target population who screen negative for anxiety problems 12 months post-randomisation (primary clinical endpoint) in schools allocated to ‘screening and intervention’ versus ‘usual school practice’. Secondary objectives are as follows:

- To compare outcomes related to anxiety, depression and behavioural problems at 4 months, 12 months and 24 months post-randomisation for children in our target population in schools allocated to ‘screening and intervention’ versus ‘usual school practice’;
- To compare school attendance and academic attainment up to the end of Year 6 (aged 10–11) for children in the target population in schools allocated to ‘screening and intervention’ versus ‘usual school practice’;
- To estimate the cost-effectiveness of ‘screening and intervention’ compared to ‘usual school practice’, extrapolating results up to 5 years beyond the duration of the trial;
- To evaluate experiences of procedures for screening, feedback and intervention to inform an integrated process evaluation;
- To compare outcomes related to anxiety, depression and behavioural problems for the total population of children (aged 8–9) in participating classes in schools allocated to ‘screening and intervention’ versus ‘usual school practice’ at 4 months, 12 months and 24 months post-randomisation.

This protocol follows the SPIRIT (Standard Protocol Items: Recommendations for Interventional Trials) reporting guidance (see Supplement 1 for SPIRIT checklist).

## Trial design

The design will be a parallel-group, superiority cluster randomised controlled trial, with an internal pilot. We will recruit schools in two cohorts/phases (pilot phase (Phase 1): target 30 schools; Phase 2: target 50 schools). An independent programme steering committee will assess continuation criteria for progressing beyond the pilot phase. All children in Year 4 (aged 8–9) in sampled classes where parents do not opt out will enrol in the trial (total population), and children, parents/carers and class teachers will be invited to complete baseline measures. Children who screen positive at baseline on the basis of a brief parent-report child anxiety screening questionnaire (iCATS-2) will be the target population. After baseline assessments are complete in a cohort of schools, schools (clusters) will be ordered by the number of children in the target population and randomised to ‘screening and intervention’ (intervention arm) or ‘usual school practice’ (control arm) in a 1:1 ratio stratified according to level of deprivation.

In schools allocated to ‘screening and intervention’: (1) Parents will complete the iCATS-2 screening questionnaire (at baseline) and will receive feedback on whether their screening questionnaire responses indicate their child may be experiencing difficulties with anxiety (screen positive) or is unlikely to be experiencing difficulties with anxiety (screen negative). (2) Children will receive a whole-class lesson on managing fears and worries, and school staff will receive information and guidance on the intervention approach and strategies. (3) Where children screen positive (target population), families will be offered a brief parent-led online intervention (OSI). OSI will also be made available to any other participating children (total population) where parents request it. Follow-up measures will be collected for the target population and the total population at 4 months, 12 months and 24 months post-randomisation. Qualitative interviews will be conducted up to around 6 months after intervention delivery. Participant recruitment will take place between January and November 2022, and data collection is expected to continue until December 2024.

## Methods

### Study setting

The study setting is mainstream primary/junior schools in England with at least two classes of Year 4 children (aged 8–9 years), and a minimum of 40 Year 4 children on the school roll. Schools with a ‘Mental Health Support Team’ (MHST) as part of the recent initiative led by the Department of Health and Social Care, Department for Education and NHS England [23] in place at the point of recruitment will be excluded. A core function of MHSTs is to deliver evidence-based mental health interventions so the trial team simultaneously delivering procedures for identification-to-intervention within these schools could create confusion for families and staff, and as MHSTs are currently only established in a minority of primary schools [23], this provision is not reflective of current usual school provision and including these schools could hinder interpretation of trial outcomes.

Single or mixed-year group classes with Year 4 pupils on the register (Year 4 classes) will take part. Where schools have two or three Year 4 classes, all Year 4 classes will take part; where schools have more than three Year 4 classes, three Year 4 classes will be randomly sampled to take part.

### Eligibility criteria

#### Inclusion criteria for the total population

##### Children

- Child is in Year 4 (aged 8–9) in a participating class. The child needs to be on the class register during the recruitment and baseline data collection period, up to the point of school randomisation.
- Child’s parent/carer does not opt the child out.

##### Parents/carers

- Parent/carer of a child in Year 4 (aged 8–9) in a participating class, and they provide written consent. Families will be asked to nominate one parent/carer to complete parent-report questionnaires for a child.

##### Teachers

- Baseline: Current class teacher or member of school staff who works regularly with children in a participating class.
- Follow-up: participating child’s current class teacher or member of support staff who works regularly with them.

#### Inclusion criteria for the target population

##### Children

- Child screens positive (score ≥ 3 out of 6) on the brief parent-report child anxiety screening questionnaire (iCATS-2) at baseline.

##### Parents/carers

- Parent/carer of a child who screens positive (score ≥ 3 out of 6) on the parent-report iCATS-2 questionnaire at baseline.

##### Teachers

- Current class teacher or member of school staff who works regularly with a child who screens positive (score ≥ 3 out of 6) on the parent-report iCATS-2 questionnaire at baseline.

#### Exclusion criteria for the trial (total and target population)

- Children who do not have sufficient English language or comprehension skills to complete measures, even with support, will not complete child-report questionnaires.
- Parents/carers who do not have sufficient English language or comprehension skills to provide consent, complete measures and/or take part in the intervention, even with support and/or translated study information, will not take part.

#### Inclusion criteria for the qualitative interviews

##### Children

- Child is in Year 4 in a participating class in a school allocated to ‘screening and intervention’.
- Child’s parent/carer provides written consent, including consent for audio-recording the interview, and child provides assent.

##### Parents/carers

- Parent/carer of child in Year 4 in a participating class in a school allocated to ‘screening and intervention’ and they provide written consent, including consent for audio-recording the interview.

##### School staff

- Member of staff in a participating school that is allocated to ‘screening and intervention’, and they provide written consent, including consent for audio-recording the interview.

##### Other key stakeholders

- Governor or representative of another key stakeholder organisation who has a professional role within or related to a participating school that is allocated to ‘screening and intervention’, for example a mental health service provider, local authority, local policy maker organisations (e.g. local public health team), and they provide written consent, including consent for audio-recording the interview.

##### Clinical members of the research team involved in delivering the intervention

- CWP or clinical psychologist who delivered and/or supervised the delivery of OSI as part of the trial, and they provide written consent, including consent for audio-recording.

#### Eligibility criteria for intervention (OSI) participants

- Parent/carer of a Year 4 child in a participating class in a school randomised to ‘screening and intervention’, and they did not opt the child out of the study.
- Parent/carer’s child screened positive (score ≥ 3 out of 6) on the parent-report iCATS-2 questionnaire at baseline and/or the parent/carer decides to take up the intervention offer and provides written consent.
- As the intervention is parent-led, it is not feasible for families to complete the intervention for more than one child in parallel. Where more than one child in a household or family takes part in the trial, the parent/carer will only be able to receive the intervention for one child (although they can apply strategies with other children in the family) and will be able to request which child receives the intervention.

### Recruitment

Schools and families will be recruited in two phases, and we expect to start baseline data collection for each phase over two consecutive academic years.

#### School recruitment

Our aim is to recruit primary/junior schools from at least four geographic regions in England that vary in relation to the demographic profile of registered pupils (percentage of pupils eligible for free school meals, percentage of pupils with English as an additional language, percentage of pupils on special education needs support). To recruit schools, we will follow a similar approach to what we have used previously to successfully recruit a large number of primary schools [10, 24]. We will identify potential schools to approach using publicly available information provided by the Department for Education, and contact school staff via email and follow-up telephone/video-calls. Information about the study will also be distributed via the team’s existing networks and social media. As school recruitment progresses, we will closely monitor the characteristics of recruited schools, and approach schools with particular characteristics as needed so that as far as possible the final sample of schools is representative of primary/junior schools in England.

Schools’ headteachers will provide written agreement (online or on paper) and we will ask schools to nominate at least two ‘iCATS leads’ to collaborate with the research team to distribute study information and coordinate study activities in the school. Where schools have more than three eligible Year 4 classes, three Year 4 classes will be randomly selected to participate.

#### Participant recruitment

Study information will be distributed to all parents/carers, Year 4 children, and staff in participating classes using a range of methods, including displaying study posters/banners at school; researchers running in-person/online information sessions; distributing video adverts and video-versions of study information; distributing paper and electronic versions of study materials for parents; advertising the study on the school website/newsletter/Twitter/Facebook; recruiting school ‘parent champions’ to help disseminate study information to other parents/carers; distributing stickers and colouring sheets with the study logo; sending reminders via SMS/email. Written study information for parents/carers will be available in English and at least four other languages that are common first languages in England (Bengali, Punjabi, Gujarati, Urdu) [25]. We will work closely with school ‘iCATS leads’ to adapt strategies for each individual school as needed to help maximise recruitment within each school.

Parents/carers will have an opportunity to opt their child out of the study. School staff will be asked to provide the research team with a list of the names of all Year 4 children in participating classes where the parent does not opt out and all these children, and their parents/carers, and class teachers will be invited to take part and complete baseline questionnaires. Prior to completing questionnaires, children will provide written assent (on paper or online), and parents/carers and teachers will provide written informed consent (on paper or online).

### Data collection procedures

**Fig. 1:**
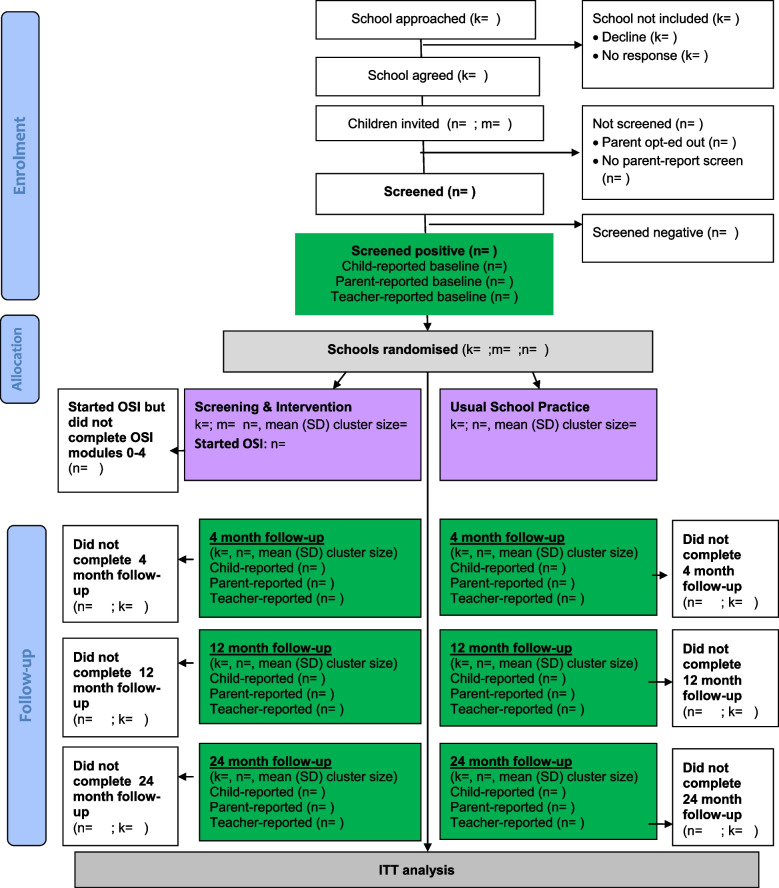
Target population: Consolidated Standards for Reporting Trials diagram. *Note*: k = number of schools; m = number of classes; n = number of children; cluster = school

**Fig. 2:**
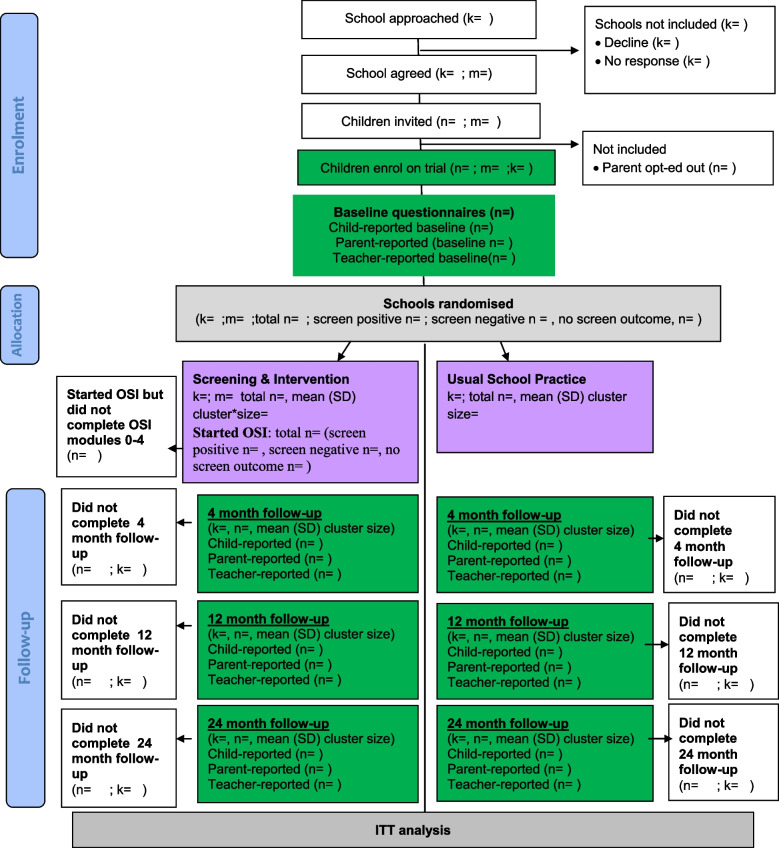
Total population: Consolidated Standards for Reporting Trials diagram. *Note*: k = number of schools; m = number of classes; n = number of children; cluster = school

An overview of study procedures and assessments are provided in Figs. 1, and 2, and Supplement 2.

#### Baseline

Parents/carers can opt to complete baseline parent-report questionnaires on paper (and return to school for the study team to collect) or online. Schools will distribute initial parent paper packs (including the consent form, contact details form, socio-demographic form, brief Spence Children’s Anxiety Scale (SCAS-8-P), and the brief child anxiety screening questionnaire (iCATS-2)), and a link where parents can complete the consent form online and receive a personalised link to all online parent-report baseline questionnaires. Once parents/carers provide written consent and their contact details (on paper or online), the study team will contact them to arrange a short ‘welcome’ call to discuss the study, answer any questions and where needed remind parents/carers to complete any outstanding questionnaires. Where parents/carers request to complete any outstanding baseline questionnaires on paper, the study team will send these directly to the family’s home address, together with a return envelope.

Researchers will visit schools to administer baseline child-report questionnaires (on paper) with each participating class, and children will be able to request to complete the questionnaires at home (on paper or online) if they prefer. Children whose parents/carers opt them out and those who decide not to complete child-report questionnaires will be given an alternative activity (e.g. colouring-in activity). Where it is not possible for researchers to administer questionnaires at schools (e.g. COVID-19-related restrictions), we will work with schools to adapt these procedures (e.g. school staff will administer child-report questionnaires at school, some or all children will complete questionnaires at home).

Class teachers (or support staff who work regularly with participating children) will be asked to complete teacher-report questionnaires (online or on paper) about each participating child in their class. We will also ask nominated members of school staff to provide information (online or via a telephone/video-call) on activities related to social, emotional and mental health and wellbeing offered for pupils in Years 4–6 in their school, keep a record of time spent on study activities and provide demographic and attendance information about participating children.

#### Intervention period

Parents/carers in schools allocated to ‘screening and intervention’ who take up OSI will complete questionnaire measures built into each online module (see below). CWPs and their supervisors will be asked to complete bespoke logs to record time spent on activities related to screening feedback and intervention delivery.

#### Follow-ups

We will collect follow-up measures at 4 months, 12 months and 24 months post-randomisation for all children enrolled on the study (total population and target population). Where parents consented and provided their contact details previously, parent-report follow-up questionnaires will be sent directly to parents/carers, either via an online survey or as paper versions sent to their home address or via school. At each follow-up assessment point, parents/carers of children enrolled on the study who did not consent/provide the study team with their contact details will also have the opportunity to consent and complete follow-up questionnaires. Where possible, researchers will visit schools to administer child-report questionnaires at each assessment point, with the option for children to complete measures at home if they prefer or researchers are not able to visit the school (e.g. COVID-19 related restrictions). At each follow-up, children who did not previously assent to complete child-report questionnaires will have the opportunity to provide assent and complete follow-up child-report questionnaires. Follow-up teacher-report questionnaires will be collected from the child’s current class teacher/member of support staff who works regularly with the child, and at each follow-up, nominated members of school staff will provide information on activities related to social, emotional and mental health and wellbeing offered in their school for pupils in Years 4–6 and provide information on children’s school attendance.

#### Qualitative interviews

We will conduct one-to-one qualitative interviews to explore experiences related to procedures for screening, feedback and intervention. Interviews will be conducted with parents/carers, children, school staff and other key stakeholders in schools allocated to ‘screening and intervention’, and clinical research team members involved in delivering feedback and the intervention. We anticipate this will include interviews with up to 20 parents (including parents of children who screen negative and screen positive, and those who do and do not take up the intervention), up to 20 children (including children who screen negative and screen positive), up to 10 members of school staff/other key stakeholders, and approximately 5 CWPs/clinical psychologists who deliver OSI as part of the trial.

#### Plans to promote participant retention and complete follow-up

At each time point, families will be offered a £10 gift voucher for completion of questionnaire measures, and teachers will be offered £100 for completion of teacher-report questionnaires for all participating children in their class, and qualitative interview participants will be offered an additional £10 gift voucher. At each school visit/assessment, children will also be offered additional incentives branded with the study logo, such as a participant certificate, fridge magnet, stickers and colouring sheets. Parents/carers who take up OSI will be offered a £30 voucher to purchase internet data they will use to work through the online programme. Each school will be offered £100 at each time point for time spent on all recruitment and data collection activities, and schools allocated to ‘usual school practice’ will receive a £1000 thank you payment at the end of the study.

After school randomisation, parents will receive a letter and follow-up telephone call from a member of the study team to remind them of the next steps and answer any questions. Throughout the 2-year follow-up period, we will send schools and families regular study updates, and at each assessment point we will use reminder emails/SMSs/telephone calls. If a school withdraws from the trial during the follow-up period, we will continue to collect follow-up measures from children and parents/carers. Where a child moves schools during the follow-up period, it will not be possible to collect follow-up information and data from the child’s new school or class teacher, but the child will remain in the study and where possible we will continue to collect follow-up child-report and parent-report measures (including the primary outcome measure). Where parents discontinue the online intervention, we will encourage them to stay in the trial and complete follow-up questionnaire measures.

### Sample size

Our target sample size is 398 children in the target population (screen positive for anxiety problems at baseline) from 80 schools. This sample size is large enough to detect an increase in the remission of anxiety problems from 50% (‘usual school practice’ arm) to 70% (‘screening and intervention’ arm). Seventy percent remission is similar to treatment outcomes achieved 6 months after receiving brief, parent-led cognitive behavioural therapy (CBT) within primary child and adolescent mental health services [20]. Fifty percent is a conservative estimate of natural remission over 12 months based on data showing 50% remission in anxiety disorders in children (aged 9–11 years) in community settings over a 2-year period [7].

The sample size is based on the following assumptions: (i) 2 or 3 classes (average 2.3 classes) per school will participate and an estimated 27 children per class (4968 children in total) will be invited, (ii) 40% of parents/carers will consent and complete the screening questionnaire (*n* = 1988), (iii) 20% of children screened will screen positive (target population) (*n* = 398), and (iv) 80% of the target population (*n* = 318) will be retained at 12 months post-randomisation. The 20% screen positive rate is in keeping with findings in our feasibility study (21% screened positive), and 40% parent participation and 80% retention reflect a modest improvement on recruitment and retention rates observed in our feasibility study (30% parent participation, 78% retention to 12 weeks). Given that our feasibility study coincided with substantial COVID-19-related restrictions and disruptions, which very likely had a negative impact on recruitment/retention, it is reasonable to expect a modest improvement on recruitment/retention rates. Equally, although our feasibility study only assessed retention to 12-week follow, in a recent UK school-based trial, 95% of parents who completed assessments immediately post-intervention were retained to 18-month follow-up [26]. The sample size also allows for clustering within schools, assuming an intra-cluster (intra-school) correlation coefficient (ICC) of 0.05 for the primary outcome, that the mean number of children retained to 12 months (cluster size) is 3.98, and allows for a coefficient of variation of the cluster size as large as 0.4; the design effect is 1.15. The median ICCs reported in a recent systematic review of school-based cluster randomised trials was 0.028, indicating that our assumed value is on the conservative side [27]. As we are recruiting schools and participants in two phases, we will also have an opportunity to review sample size assumptions following the pilot phase, and consider adjusting the total number of schools and/or classes to help ensure we recruit a sufficient numbers of participants in the target population.

The total population will include: (1) children who screen positive for anxiety problems, (2) children who screen negative for anxiety problems and (3) children with no screening outcome (i.e. no parent-report baseline screening questionnaire). As the target population is our primary focus and a subset of the total population, our formal recruitment targets relate specifically to this target group; however, we expect parents/carers of approximately 10% of invited children will opt out, providing an estimated 4471 children in the total population, with variation in questionnaire completion rates across reporters.

### Assignment of interventions: allocation and blinding

Schools will be randomised to ‘screening and intervention’ or ‘usual school practice’ in a 1:1 ratio stratified according to level of deprivation among pupils on the school roll (above/below 2020/2021 national average of 21.6% of pupils eligible for free school meals [28]). All schools in a phase will be randomised en bloc, after baseline assessments have been completed. Prior to allocation, schools in each phase will be ordered according to the number of children in the target population. An independent statistician will conduct randomisation using a computer-generated allocation sequence with block sizes of two and four in order to minimise imbalance in the sample size between the trial arms. The independent statistician will pass the allocation to the study team who will inform schools and parents/carers which arm they are allocated to.

Given the nature of the intervention, it is not possible to blind schools, participants or CWPs/supervisors to allocation. Primary and secondary outcome measures are self-completed questionnaires (child-, parent-, teacher-report) which minimises the risk of assessor bias. A statistical analysis plan and health economic analysis plan will be written and signed off prior to conducting any analyses, and statisticians/health economists will remain blind to allocation up until the point of receiving the final datasets for analysis.

### Interventions

#### Screening and intervention

##### Feedback for parents/carers on screening outcomes

Where parents/carers complete the baseline screening questionnaire and provide their contact details, they will receive a feedback letter letting them know if their responses indicate their child may be experiencing anxiety problems (screen positive) or is unlikely to be experiencing anxiety problems (screen negative). The letter also provides information about the online intervention programme (OSI). Where children screen positive, we will contact parents/carers to arrange a short feedback call with a CWP where they have the opportunity to discuss the screening outcome and are offered OSI. Where children screen negative, the feedback letter explains that the intervention programme is available for all parents/carers who feel their child may benefit, regardless of initial questionnaire responses, and parents/carers are invited to get in touch with the study team should they wish to discuss the intervention. School staff will also distribute information to all parents/carers of Year 4 children in participating classes to remind them that the intervention is available for any families (who did not opt-out) who request it, including those where parents/carers did not complete initial questionnaires. We will also provide school staff with the names of children who screen positive, where parents/carers consent to us sharing this information.

##### Class lesson on recognising and managing anxiety and resources for school staff

The research team and/or school staff will deliver an interactive whole-class lesson (approximately 60 min) on recognising and managing fears, worries and anxiety for each participating class (total population). School staff will be provided with lesson materials (slideshow with accompanying audio, and materials for interactive activities) to reuse and/or provide for other classes within the school. We will also share information and guidance about the intervention and skills and strategies parents/carers learn with school staff, including written information and a slideshow presentation with accompanying audio.

##### Online Support and Intervention for child anxiety (OSI)

Where parents agree to take up the intervention offer, they will be given access to the OSI parent website. OSI is an online version of an evidence-based face-to-face parent-led treatment for child anxiety disorders [19, 20] that was developed through a process of user-centred design and usability testing [21]. We adapted the content of OSI for delivery through primary schools following procedures for universal screening [17]. Parents work through a series of 7 weekly online modules (Modules 0–6), each supported by a short telephone call (approximately 20 min) with a CWP, and a follow-up review about 4 weeks after the intervention is completed (Module 7). Each online module takes about 20–30 min and includes inbuilt routine outcome questionnaire measures, simple text, videos and animations to explain strategies, interactive activities (e.g. questions and module quiz) and audio versions of module content. Online modules teach parents skills and strategies to apply in their child’s daily life, including ways to explore their child’s anxious thoughts, test these thoughts by facing fears (exposure), problem solve challenges and develop independence. Parents/carers will also be given access to an accompanying, optional mobile game app (Monster’s Journey: Facing Fears) for their child which is designed to help motivate children to engage with the intervention activities. At the end of the intervention, parents receive a written report summarising their child’s progress and with parental permission this report will also be shared with the child’s GP/school/another professional or service. Parents/carers who complete a minimum of the first five online modules (defined as completing the inbuilt questionnaire measures presented at the start of Modules 0–4) will have received the key intervention content and will be categorised as a ‘complier’ for the purpose of complier analyses detailed below. More information about OSI and screenshot images are available here: https://osiresearch.org.uk/osi/

We anticipate that at least four CWPs who are trained to deliver low-intensity psychological interventions for children and young people will support OSI delivery for the trial. The CWPs will receive initial training in parent-led CBT for child anxiety problems and OSI specifically, through reading, discussion, observation and role-play, and will follow highly structured and standardised guidance on how to support parents to work through the online programme, practice strategies and problem solve difficulties. CWPs will receive weekly supervision from a clinical psychologist with expertise in parent-led CBT for child anxiety problems and OSI specifically. Supervising clinical psychologists will closely monitor intervention adherence throughout, and with parent consent, telephone sessions will be audio-recorded for use in supervision.

#### Usual school practice

We aim to determine if procedures for screening, feedback and intervention for child anxiety problems bring clinical and health economic benefits above and beyond current school provision. Families in both arms will be free to continue to seek and access any usual care or support for anxiety or other difficulties, and schools will continue to provide any usual social, emotional and mental health support and intervention for children and families. At the end of the trial (after the 24-month assessment), families in schools allocated to ‘usual school practice’ will be offered written (PDF) versions of the content of the online programme, and these schools will receive the accompanying information about the intervention strategies and materials for the class lesson on managing fears and worries.

### Screening and outcome measures

#### Screening measure

The brief parent-report child anxiety screening questionnaire (iCATS-2) will be used to assess child anxiety problems at baseline and identify the target population. The iCATS-2 consists of two items that assess the extent to which a child’s fears, worries or anxiety cause distress (*Do your child’s fears, worries or anxiety upset or distress your child?*) and interfere with family life (*Do your child’s fears, worries or anxiety make things difficult for your family as a whole?*). Parents rate each item on a 4-point scale (No, not at all = 0; Yes, only a little = 1, Yes, quite a lot = 2; Yes, a great deal = 3), with responses summed to provide a total score and binary outcome (a total score of 3 to 6 is a positive screen and a score of 0 to 2 is a negative screen). The cut-off score of ≥ 3 identifies children (aged 8–11) with anxiety disorders with 76% sensitivity and 80% specificity. Children who screen positive (total score ≥ 3) at baseline will be the target population.

#### Primary outcome measure

Our primary outcome is the proportion of children in the target population who screen negative for child anxiety problems (score 0–2 on the parent-report iCATS-2 questionnaire) at 12 months post-randomisation.

#### Secondary clinical outcome measures

We will assess child anxiety problems using the parent-report iCATS-2 questionnaire at each assessment point in both the target population and total population, providing a secondary binary outcome (screen positive/screen negative), at 4 months (target and total population), 12 months (total population), and 24 months post-randomisation (target and total population).

We will also assess the following child clinical outcomes in both the target population and total population at baseline, 4 months, 12 months and 24 months post-randomisation:

- *Child anxiety symptoms* will be measured using the child-, parent- and teacher-report versions of the brief Spence Children’s Anxiety Scale (SCAS-8-C/P/T) [29] and the child- and parent-report versions of the Revised Children Anxiety and Depression Scale (RCADS-C/P)-Anxiety Scale [30]. The SCAS-8 consists of 8 items from the original SCAS that are able to discriminate between children (aged 7–11) in a community sample and clinic-referred children with anxiety disorders [29]. Items are rated on a 4-point scale (Never = 0, Sometimes = 1, Often = 2, Always = 3) and summed to provide a total score (0–24). With child-, parent- and teacher-report versions available, the SCAS-8 will provide a consistent measure of child anxiety symptoms across the three reporters. The RCADS-C/P is a well-established measure of child anxiety and depression symptoms that is widely used in clinical services [31] and evaluations of school-based interventions for child anxiety [32]. The Anxiety scale items were mostly derived from the original SCAS and includes 37 items that are rated on a 4-point scale (Never = 0, Sometimes = 1, Often = 2, Always = 3), and summed to produce a total Anxiety score (0–111). Seven items that appear on the SCAS-8-C/P also appear on the RCADS-C/P.
- *Interference related to child anxiety* will be assessed using additional items presented alongside the SCAS-8 for each reporter. Parents will complete the iCATS-2 as detailed above, and children and teachers will complete the following corresponding items rated on a 4-point scale (0–3): Children: *Do fears or worries upset you?; Do fears or worries stop you from doing things?; Do your fears or worries make things difficult for people around you (e.g. family, friends, teachers)?* (total score 0–9); Teachers: *Do fears, worries or anxiety upset or distress this child?; Do this child’s fears, worries or anxiety make things difficult for you or the class as a whole?* (total score 0–6). Multi-reporter interference measures are valued by families [33], but existing measures are long (> 25 items) and developed for clinical populations, thus limiting their suitability for community populations that include children without elevated anxiety symptoms. We have previously found that child- and teacher-reported items that assess anxiety-related interference do not discriminate between children with and without anxiety disorders in a community population with sufficient accuracy for screening purposes. However, our co-design work indicates that where possible it is important to capture child, parent and teacher perspectives for key measures [17] so we will include corresponding items that assess interference related to child anxiety for each reporter to provide a multi-reporter perspective.
- *Child depression symptoms* will be assessed using child- and parent-report versions of the 10-item Revised Children Anxiety and Depression Scale (RCADS-C/P)-Depression Scale. Items are responded to on a 4-point scale (0–3) and summed to produce a total Depression score (0–30).
- *Child behavioural problems* will be measured using the conduct problems and hyperactivity/inattention subscales of the child- and parent-report versions of the Strengths and Difficulties Questionnaire (SDQ-C/P-conduct problems, 5 items, possible score 0–10; SDQ-C/P-hyperactivity/inattention, 5 items, possible score 0–10) [34]. The SDQ is widely used in community settings, with strong support for the psychometric properties of the conduct problems and hyperactivity/inattention subscale scores [35].

#### Learning-related outcomes

School staff will provide information on school attendance from the school records for the target and total population at each assessment point (baseline, 4 months, 12 months and 24 months post-randomisation) and subject to approval from the Department for Education, we plan to collect information on school attendance (Years 4–6) and academic attainment (Key Stage 2 English and Maths National curriculum assessment outcomes) from the National Pupil Database.

#### Health economic outcomes

- C*hild health-related quality of life* will be assessed using the child- and parent-report versions of the Child Health Utility-9D (CHU-9-C/P) [36, 37], and of the EQ-5D-Y (EQ-5D-Y-C/P) [38, 39]. The CHU-9D measure includes nine dimensions (worried, sad, pain, tired, annoyed, schoolwork, sleep, daily routine, activities) each with five ordered levels and was originally developed and validated with children aged 7–11 years. The EQ-5D-Y measure includes five dimensions (mobility, looking after myself, doing usual activities, having pain or discomfort and feeling worried, sad or unhappy) each with three ordered levels. Both are generic, preference-based measures of health-related quality of life in children and young people, and both allow the calculation of quality-adjusted life years (QALYs) for use in cost-utility analysis [40]. *Parent health-related quality of life* will be assessed using the parent self-reported EQ-5D-5L [41], a well-validated, generic, preference-based measure used in adult populations, designed to estimate QALYs, and widely used across disease areas. It includes five dimensions covering domains of everyday life, i.e. mobility, self-care, usual activities, pain/discomfort and anxiety/depression, each with five ordered levels of response. These measures will be collected at baseline, 4 months, 12 months and 24 months post-randomisation (target and total population).
- *Individual health and social care and broader resources* used by both the child and parent during the trial period (e.g. health and social care use, including mental health service use; time off school (child) and time off work (parent), when they are related to child anxiety problems), will be measured using a modified version of the Client Services Receipt Inventory (CSRI) [42]. Parents will complete the CSRI at baseline (with reference to the previous 3 months), 4 months, 12 months, 24 months post-randomisation, and at each assessment point will be provided with a diary to keep a record of use of services and time off work/school to facilitate completion of the CSRI at the subsequent assessment.
- To measure resources used in screening, feedback and intervention, school staff, CWPs and supervisors will complete bespoke logs to record time spent on activities related to these procedures. Information relates to OSI usage, including time parents spend on online modules (see below), will be captured within the OSI platform.

#### Additional information and measures

School-level demographic information (local education authority area, number of pupils on the roll, percentage of pupils eligible for free school meals, percentage of pupils on special educational needs support, percentage of pupils with English as an additional language) will be collected from publicly available information provided by the Department for Education.

Child demographic information will be collected from the child’s school records (gender, ethnicity, eligibility for free school meals, English as an additional or first language, information on any special education needs and Education Health and Care Plan), and parents/carers who consent will also be asked to provide socio-demographic information about their child (date of birth, gender, ethnicity), themselves (age, gender, ethnicity, relationship to child, whether they have a partner) and their household (parent highest level of education, parent and partner employment status, parent occupation, income, postcode, housing tenure, number of children and adults living in household). Class teachers/support staff that complete teacher-report questionnaires for their class will provide some background information about themselves (age, gender, ethnicity, role, number of years teaching experience).

#### Usual school practice

Nominated members of school staff will complete a bespoke questionnaire (via an online survey or telephone/video-call interview) at each baseline, and 12 months and 24 months post-randomisation to provide information on activities related to social, emotional, mental health and wellbeing provided for children in Years 4 to 6 and their families. Survey questions are open-ended and based on similar questions used in previous school-based trials [24], and we will use responses collected at baseline to identify additions or refinements needed for questions used at follow-up. We plan to analyse open-ended responses using a content-analysis approach to provide a description of provision within each school. If meaningful to do so, we will categorise descriptions according to the extent of provision (e.g. minimal, moderate, extensive).

#### Acceptability

Children, parents/carers and class teachers will complete a bespoke questionnaire to assess acceptability of study procedures at each assessment point. The measure for each reporter includes 7 items (parent and teacher versions rated on 5-point scale (1–5) from completely disagree to completely agree; child version rated on 3-point scale (1–3), not true-a little bit true-true), including items that assess positive experiences (e.g. ‘Taking part in the study was helpful for me and/or my child’) and negative/adverse experiences (e.g. ‘Taking part in the study was harmful for me and/or my child’).

#### Additional measures for the ‘screening and intervention’ arm

##### Experiences of procedures

We will use topic-guided interviews tailored for each participant group, and interviews will be audio-recorded. Full details related to qualitative interviews will be provided in a separate process evaluation protocol.

##### Measures to guide OSI and OSI usage data

Parents/carers who access OSI will complete questionnaire measures built into each online module, including measures of anxiety and depressive symptoms (RCADS-P, a ‘tracked subscale’ from the RCADS-P that best captures child’s primary problem, and the SCAS-8-P), anxiety-related interference (Child Anxiety Impact Scale) [43], overall functioning (Child Outcome Rating Scale) [44], progress against intervention goals (Goal Based Outcomes) [45] and therapeutic alliance (Session Rating Scale) [46]. Usage data captured within the online programme will also be collected, including device used to access the programme, modules completed, optional activities completed (e.g. completion of module questions and quizzes), time spent on module pages and number of times module pages are viewed.

### Progression criteria

Criteria for progressing to Phase 2 of the trial are detailed in Supplement 3. Criteria relate to the number of schools recruited in the pilot phase, participant recruitment and 4-month post-randomisation retention rates for the target population, and adverse events related to participation in the pilot phase. The independent steering committee will review progression criteria after the 4-month follow-up assessment is complete for the pilot phase, and make a recommendation about whether to continue to participant recruitment for Phase 2.

### Planned analysis

#### Primary and secondary clinical outcomes

Full details of the analyses will be specified in a separate statistical analysis plan. Characteristics of schools (clusters), families and children (target and total populations) will be summarised by trial arm status and overall, using means and SDs (or medians and interquartile ranges) for continuous variables and numbers and percentages for categorical variables. Baseline measures of child-, parent- and teacher-report child anxiety symptoms (SCAS-8) and impact items, and child- and parent-report child anxiety symptoms (RCADS anxiety score), depression symptoms (RCADS depression score) and behavioural problems (SDQ conduct problems and hyperactivity/inattention subscales) will also be summarised.

The primary analyses of all outcomes will be based on imputed data, under the intention-to-treat (ITT) principle where participants are analysed according to the trial arm to which they were randomised. Binary outcomes, including the primary outcome (absence of child anxiety problems on the basis of the parent-report iCATS-2), will be compared between trial arms using marginal logistic regression models using Generalised Estimating Equations (GEEs) [47] with information sandwich (‘robust’) standard errors, assuming an exchangeable correlation structure. Continuous outcomes will be compared using mixed effects linear regression, including random effects at the school (cluster) level to allow for the correlation between measures from the same cluster. Ordinal outcomes will be compared between trial arms using ordinal logistic regression with information sandwich (‘robust’) standard errors, provided there is no clear evidence against the assumption of proportional odds. Otherwise, they will be dichotomised and analysed as binary outcomes, as described above. For the total population, outcomes will be analysed for all children and for those children who screen negative at baseline. Crude (unadjusted) and adjusted analyses will be undertaken. Adjusted analyses will be adjusted for baseline value of the outcome (for continuous outcomes, where collected); child-level free school meal status (as a binary variable); school-level free school meal status (as a binary variable, the stratification variable); cluster size (as a continuous variable—i.e., the number of enrolled pupils in the target population, used in the randomisation process) and cohort/phase status. Analysis of the parent-report iCATS-2 (binary outcome) in the total population will additionally be adjusted for the school (cluster) level percentage of pupils who screened positive on the questionnaire at baseline (out of those children for whom a questionnaire was completed). For binary outcomes, results will be reported as the total number of children analysed in each trial arm, the number and percentage with the outcome of interest in each trial arm, unadjusted odds ratio (OR) and adjusted OR with 95% CI and *p*-value. Additionally, for the primary outcome at 12 months post-randomisation, an unadjusted risk difference with 95% CI will be obtained by specifying the identity link function instead of the logit link. For ordinal outcomes, results will be reported as the total number of children analysed in each trial arm, the number and percentage in each category of the outcome in each trial arm, unadjusted OR and adjusted OR with 95% CI and *p*-value. For continuous outcomes, results will be reported as the total number of children analysed in each trial arm, the mean and SD of the outcome of interest in each trial arm, unadjusted mean difference and adjusted mean difference with 95% CI and *p*-value. ICCs from unadjusted analyses will be reported for all outcomes.

The adjusted analyses will be considered the main analyses. Analyses based on the complete case data will also be conducted (ITT, adjusted and unadjusted) as sensitivity analyses, as well as analysis of the primary outcome and each of the secondary outcomes, including outcome data collected outside the pre-specified data collection windows (+ / − 1 month). A complier average causal effect (CACE) analysis will be undertaken of the primary outcome, absence of anxiety problems in the target population at 12 months post-randomisation, with compliance defined as completing a minimum of the first five online modules (Modules 0 to 4), as defined above. Findings will be presented in accordance with the CONSORT extension for cluster randomised trials [48].

#### Health economic analysis

Details of the economic aspects of the study will be fully described in a health economics analysis plan (HEAP) [49], which will be finalised before any analysis takes place.

The main economic evaluation will be conducted in relation to the target population to establish whether ‘screening and intervention’ is good value for money compared to ‘usual school practice’. It will comprise cost-utility (CUA) and cost-effectiveness (CEA) analyses from the NHS and Personal Social Services (PSS) perspective (primary analysis) [50], adopting an intention-to-treat approach. A secondary analysis will take a societal perspective, acknowledging that the economic costs of mental health problems have wide consequences beyond the health and social care sectors, including lost education for children, and productivity losses for parents. Best-practice guidelines for conducting economic evaluations and reporting results will be adhered to [50, 51]. The primary economic analysis will compare the costs and consequences of each trial arm at 24-month follow-up (within-trial/short-term economic evaluation). To explore the medium-term implications of ‘screening and intervention’, a secondary economic analysis will use decision analytic modelling methods [52] to extrapolate RCT data up to 5 years post-randomisation (medium-term economic evaluation) by supplementing them with relevant existing secondary data (e.g. Millennium Cohort Study [53]) and data from the literature. Longer time horizons (up to 10 years) may be explored in scenario analyses, conditional on data availability. An interim economic analysis will be conducted at the 12-month follow-up in alignment with the primary clinical endpoint. Costs and consequences in the primary and secondary economic analyses, but not in the interim analysis, will be discounted to present values using the 3.5% discount rate recommended by the National Institute for Health and Care Excellence (NICE) [50]. Multiple imputation methods will be adopted to deal with missing data [54, 55] and, in line with the statistical analyses, both primary and secondary economic analyses will be based on imputed data. In the CUA, reported health outcomes will be QALYs gained for the child, as derived from the CHU-9D and the EQ-5D-Y (child-report in primary and secondary analyses), and QALYs gained for the parent, as derived from the EQ-5D-5L, combined with QALYs gained for the child (in sensitivity analyses). In the CEA, the primary clinical outcome will be used, i.e. the proportion of children in the target population who screen negative for child anxiety problems (score 0–2 on the parent-report iCATS-2 questionnaire) at 12 months post-randomisation. For each child, treatment use (as applicable), other health and social care use, and further individual, family and wider societal costs will be collected using the CSRI questionnaire [42] completed by the parent, and the bespoke logs completed by CWPs/supervisors/school staff. Costs will be computed by multiplying units of resource use by their unit costs and then summed to obtain a total cost per child in the target population. Unit costs for health, social care, and other resources will be mainly derived from local and national sources (e.g. PSSRU [56]; National Cost collection for the NHS [57]; NASUWT [58]). Costs will be expressed in pounds sterling at current prices and adjusted for inflation as appropriate. Statistical methods for combining costs and outcomes will take account of the correlation between costs and outcomes at both the individual level and the cluster level [59]. The economic evaluation outcomes will be expressed as incremental cost per QALY gained in the CUA, and incremental cost per child free of anxiety problems in the CEA. Uncertainty around results will be accounted for and presented using cost-effectiveness acceptability curves [60]. A number of sensitivity analyses (deterministic and probabilistic) will be undertaken to explore the implications of uncertainty surrounding the incremental cost-effectiveness ratios and to consider the generalisability of the study results [52]. These will include the following: using parent-report of CHU-9D and EQ-5D-Y in estimating QALYs in the CUAs; conducting a CUA from the societal perspective where the outcome is QALYs for the parent–child dyad; conducting primary analyses on complete cases only. In the spirit of the PHE Prevention Concordat for Better Mental Health [61, 62], we will explore presenting value for money of the intervention in terms of Return On Investment (ROI) [15, 63, 64], by estimating the total costs that can be avoided to different sectors (NHS, schools, families) for every pound invested in the intervention.

Economic analyses will also be conducted in relation to the total population, and for those children who screen negative at baseline, using information collected during the trial duration (i.e. baseline, 4 months, 12 months and 24 months post-randomisation). These additional analyses will take the form of cost-consequence analyses [65], in which we will report mean QALYs/resource use/costs and their standard deviations stratified by trial arm as well as mean differences in those measures between the two trial arms alongside their 95% confidence intervals.

#### Participant experiences

Planned analysis of qualitative data related to experiences of procedures for screening, feedback and intervention will be provided in a separate process evaluation protocol.

### Data management

Full details related to data processing, checking, cleaning and storage will be specified in a separate study data management plan. We will use REDCap (Research Electronic Data Capture) databases to capture data provided by participants via online surveys and data collected on paper and manually entered by members of the research team. Data held in REDCap databases is stored on secure University of Oxford servers. Each school and participant will be assigned a unique school and participant ID, and these IDs will be used to label all study data. A document linking school/participant ID and personal details and contact information will be stored separately from other data, with access restricted to members of the study team involved in collecting data and delivering the intervention. At each assessment point, participants will be asked to confirm current contact information and child’s current school, and records updated where required. At the end of the trial, the linking document including personal details/contact information will be permanently deleted.

Pseudononymised study databases will be checked, cleaned, locked and signed off by CC and TR prior to sharing with study statisticians and health economists via restricted access OneDriveforBusiness folders. Once main trial analyses are complete, we plan to make a sufficiently anonymised version of the main study databases available in a public repository for use by other researchers.

### Oversight and monitoring

CC (PI) will oversee all aspects of the trial, and MV holds primary responsibility for health economic elements, OU for statistical analyses and ML for qualitative components. CC (PI), TR (Study Lead) and LT (Trial manager) will supervise the day-to-day running of the study and researchers based at the University of Oxford involved in data collection activities. The Study Management Group (SMG) includes all investigators and senior team members (TR, LT, VW) and will meet twice a year as a whole group, with additional subgroup meetings as needed. The Programme Steering Committee (PSC) was set up in previous phases of underpinning work and includes a chair and three additional independent members, including experts in evaluations of school-based mental health interventions, statistical methods for trials, qualitative and mixed-methods in mental health research and school-based mental health and wellbeing provision. The PSC will meet at least annually to monitor and review study progress, including to review progression criteria prior to starting phase 2 participant recruitment.

A Data Monitoring and Ethics Committee (DMEC) was set up for this trial and comprises four independent members (including a chair), with expertise in trials, statistical methods, health economic evaluations and delivery of low-intensity psychological interventions within schools. The DMEC will assess the safety of the intervention during the trial, monitor the overall conduct of the trial, have access to unblinded data as required and make recommendations to the PSC on whether there are safety or ethical reasons why the trial should not continue. It is anticipated that the DMEC will meet annually, timed so that they can make recommendations ahead of PSC meetings.

#### Adverse events reporting and harms

Study protocols for managing any potential risk or safeguarding concerns will be followed, and any potential adverse events will be recorded and monitored in line with the study adverse events protocol. Potential adverse events will be recorded, logged and monitored by the PI and SMG, and serious adverse events will be reported to the PSC and DMEC.

### Dissemination plans

We will share a summary of trial outcomes with schools and families, and disseminate findings widely to reach a range of audiences, for example we will publish outcomes in open access articles in high-quality journals to reach academic, clinical and education audiences; present findings at national and international conferences and events; share tailored reports for policy makers, and healthcare and education providers and share findings via the study website, newsletters, blogs and social media platforms.

## Discussion

This trial aims to establish the clinical and health economic benefits of incorporating screening, feedback and intervention into usual school provision, compared to current usual school provision only, for children (aged 8–9) with anxiety problems (target population) and the wider population of all children in participating classes (total population). If effective and cost-effective, our procedures for identification-to-intervention would improve access to early intervention for child anxiety problems and reduce the associated negative consequences for children, families and wider society. Our procedures and findings can also be used to inform future development and evaluation of systematic approaches to identifying children and adolescents with other mental health problems, and offering and delivering evidence-based interventions for those identified as likely to benefit in primary and secondary school settings.

## Trial status

School recruitment began in October 2021 and is expected to continue to September 2022, and participant recruitment began in January 2022 and is expected to continue to November 2022. This protocol is version 3, 31.8.2022.

## Supplementary Information

**Additional file 1: Supplement 1.** SPIRIT Checklist.

**Additional file 2: Supplement 2.** Schedule of enrolment, intervention, and assessment.

**Additional file 3: Supplement 3.** Progression criteria assessed by the Programme Steering Committee prior to progressing to Phase 2.

## Data Availability

Datasets and study materials generated during the current study will be made available in a public repository.

## Abbreviations

CACE: Complier average causal effect
CEA: Cost-effectiveness analysis
CUA: Cost-utility analysis
CSRI: Client Services Receipt Inventory
CWP: Children’s Wellbeing Practitioner
CHU-9D: Child Health Utility 9 Dimension instrument
DMEC: Data Monitoring and Ethics Committee
EQ-5D-5L: EuroQol 5 Dimension -5 Level instrument
EQ-5D-Y: EuroQol 5 Dimension-Youth version instrument
GEE: Generalised Estimating Equations
HEAP: Health Economics Analysis Plan
iCATS-i2i: Identifying Child Anxiety Through Schools-identification to intervention
iCATS-2: Brief child anxiety screening questionnaire
ICC: Intra-cluster correlation coefficient
ITT: Intention-to-treat
MHST: Mental Health Support Team’
NICE: National Institute for Health and Care Excellence
OSI: Online Support and Intervention for child anxiety
PSS: Personal Social Services
QALY: Quality-adjusted life years
RCADS: Revised Children Anxiety and Depression Scale
REDCap: Research Electronic Data Capture
SCAS: Spence Children’s Anxiety Scale
SDQ: Strengths and Difficulties Questionnaire
SMG: Study Management Group
