# Supplementary material for: Identifying Child Anxiety Through Schools-identification to intervention (iCATS-i2i): protocol for a cluster randomised controlled trial to compare screening, feedback and intervention for child anxiety problems to usual school practice
Source: Trials. 2022 Oct 22;23:896. doi: 10.1186/s13063-022-06773-0 (PMC9587579; doi:10.1186/s13063-022-06773-0)
Supplement: Supplementary file 2 — Additional file 2: Supplement 2. Schedule of enrolment, intervention, and assessment. [file 13063_2022_6773_MOESM2_ESM.docx]

Supplement 2 Schedule of enrolment, intervention, and assessment

|  | **Enrolment** | | **Allocation** | **Post allocation** | | | |  |
| --- | --- | --- | --- | --- | --- | --- | --- | --- |
|  | **School enrolment** | **Participant enrolment/ Baseline (T0)** |  | **Intervention** | **4 month assessment (T1)** | **12 month assessment (T2)** | **24 months assessment (T3)** |  |
| **Time point (months relative to randomisation)** | **--3 to -2** | **-2 to -0** | **0** | **0-4** | **4**  **(+/- 1)** | **12**  **(+/- 1)** | **24 (+/-1)** |  |
| **Enrolment** | | | | | | | | |
| School consent | X |  |  |  |  |  |  |  |
| Parent opt-out |  | X |  |  |  |  |  |  |
| Child assent |  | X |  |  | X^a^ | X^a^ | X^a^ |  |
| Parent consent |  | X |  | X^a^ | X^a^ | X^a^ | X^a^ |  |
| Teacher consent |  | X |  |  | X^a^ | X^a^ | X^a^ |  |
| Consent for qualitative interview |  |  |  | (if not provided previously) | | |  |  |
| **Allocation** | | | | | | | | |
| School randomisation |  |  | X |  | | |  |  |
| **Intervention** | | | | | | | | |
| Feedback for parents/carers |  |  |  | X |  |  |  |  |
| OSI |  |  |  | X |  |  |  |  |
| Anxiety lesson and intervention resources for school staff |  |  |  | X |  |  |  |  |
| Usual school practice (all schools) |  |  |  |  |  |  |  |  |
| **Assessments** | | | | | | | | |
| **Demographic/background and contact information** | | | | | | | | |
| School-level demographic information (DfE website) | X |  |  |  |  |  |  |  |
| Child demographic information and Unique Pupil number collected from school records |  | X |  |  |  |  |  |  |
| Family demographic information (parent-report) |  | X |  | X^a^ | X (any change) | X (any change) | X (any change) |  |
| Family contact information (parent-report) |  | X |  | X^a^ | X (any change) | X (any change) | X (any change) |  |
| Teacher demographic information |  | X |  |  | X^a^ | X^a^ | X^a^ |  |
| Qualitative interview participant demographic information |  |  |  | (if not collected previously) | | |  |  |

|  | **Enrolment** | | **Allocation** | **Post allocation** | | | |
| --- | --- | --- | --- | --- | --- | --- | --- |
|  | **School enrolment** | **Participant enrolment/ Baseline (T0)** |  | **Intervention** | **4 month assessment (T1)** | **12 month assessment (T2)** | **24 months assessment (T3)** |
| **Time point (months relative to randomisation)** | **--3 to -2** | **-2 to -0** | **0** | **0-4** | **4**  **(+/- 1)** | **12**  **(+/- 1)** | **24 (+/-1)** |
| **Child anxiety screen (presence/absence of child anxiety problems)** | | | | | | | |
| 2-item child anxiety questionnaire (parent-report) |  | X |  |  | X | X | X |
| **Broader clinical outcomes** | | | | | | | |
| Child anxiety: SCAS-8 with impact supplement (child-report, teacher-report, parent-report) |  | X |  |  | X | X | X |
| Child anxiety and depression: RCADS (child-report and parent-report) |  | X |  |  | X | X | X |
| Child behavioural problems symptoms: SDQ-conduct problems and hyperactivity/inattention subscales (child-report and parent-report) |  | X |  |  | X | X | X |
| **To guide OSI** | | | | | | | |
| RCADS (parent-report) |  |  |  | OSI Modules 0, 6 7 |  |  |  |
| RCADS-target subscale (parent- report) |  |  |  | OSI Modules 1 to 5 |  |  |  |
| CAIS (parent- report) |  |  |  | OSI Modules 0, 6, 7 |  |  |  |
| CAIS-global subscale (parent- report) |  |  |  | OSI Modules 1 to 5 |  |  |  |
| SCAS-8 (parent- report) |  |  |  | OSI Modules 0 to 7 |  |  |  |
| Goal-based outcome (parent- report) |  |  |  | OSI Modules 1 to 7 |  |  |  |
| Child Outcome Rating Scale (parent- report) |  |  |  | OSI Modules 0 to 7 |  |  |  |
| Session Rating Scale (parent-report) |  |  |  | OSI Modules 0 to 7 |  |  |  |
| **Learning related measures and outcomes** | | | | | | | |
| Child attendance and punctuality collected from school records |  | X |  |  | X | X | X |
| Child Year 6 (aged 11) academic outcomes and school attendance collected from the National Pupil Database^b^ |  |  |  |  |  |  | X^c^ |
| **Health economic measures and outcomes** | | | | | | | |
| Child health-related quality of life: CHU-9D and EQ-5D-Y (child-report and parent-report) |  | X |  |  | X | X | X |
| Parent health-related quality of life: EQ-5D-5L (parent-self-report) |  | X |  |  | X | X | X |
| Child and parent use of services: Modified Client Services Receipt Inventory and diary (parent-report) |  | X |  |  | X | X | X |
| Children’s Wellbeing Practitioner therapy and supervision log |  |  |  |  |  |  |  |
| Supervisor supervision log |  |  |  |  |  |  |  |
| School staff log (time spent on study activities) |  |  |  |  |  |  |  |
| **Additional information and measures** | | | | | | | |
| Adverse events:   - Bespoke questionnaire (child-report, parent-report, teacher-report) - Parent/child/teacher reports throughout |  | X |  |  | X | X | X |
| Acceptability: bespoke questionnaire (child-report, parent-report, teacher-report) |  | X |  |  | X | X | X |
| Experiences of iCATS-i2i procedures: One-to-one qualitative interviews |  |  |  |  |  |  |  |
| OSI usage data |  |  |  |  |  |  |  |
| School social and emotional wellbeing activities (collected from school staff) |  | X |  |  |  | X | X |

*Note.*

*^a^* collected from children/parents/teachers who did not provide assent/consent at baseline. ^b^ subject to approval from Department for Education. ^c^ anticipate data collection will take place after trial period.

T0 - Baseline assessment; T1- 4 month post-randomisation assessment; T2 - 12-month post-randomisation assessment; T3 - 24-month post-randomisation assessment; OSI - Online Support and Intervention for child anxiety; DfE - Department for Education; SCAS-8 - Brief Spence Children’s Anxiety Scale; RCADS - Revised Children’s Anxiety and Depression Scale; SDQ - Strengths and Difficulties Questionnaire; CHU-9D - Child Health Utility-9-Dimension; EQ-5D-Y - EuroQol --Youth instrument; EQ-5D-5L - EuroQol-5-Dimension – 5-Level instrument; CSRI - Client Service Receipt Inventory. CAIS=Child Anxiety Impact Scale.
