# Supplementary material for: Identifying Child Anxiety Through Schools-identification to intervention (iCATS-i2i): protocol for a cluster randomised controlled trial to compare screening, feedback and intervention for child anxiety problems to usual school practice
Source: Trials. 2022 Oct 22;23:896. doi: 10.1186/s13063-022-06773-0 (PMC9587579; doi:10.1186/s13063-022-06773-0)
Supplement: Supplementary file 3 — Additional file 3: Supplement 3. Progression criteria assessed by the Programme Steering Committee prior to progressing to Phase 2. [file 13063_2022_6773_MOESM3_ESM.docx]

|  | **Progression criteria** |
| --- | --- |
| **GO:**  **We will continue to recruit beyond the pilot if all of the criteria below are met:** | - Recruitment of at least 24 of the planned 30 pilot schools. - At least 8% of Year 4 children in participating classes are consented and screen positive* - Retention of 80% of parents of children who screen positive at baseline at the first follow-up assessment (as a proxy for the 1-year assessment as most drop-outs occur in this first phase). - Absence of serious adverse effects that can be attributed to participation in the trial (confirmed by Programme Steering Committee). |
| **AMEND:**  **We will consult with the PSC regarding progression if (any of the following):** | - 18-23 pilot schools have been recruited. - Less than 8% but at least 6% of Year 4 children in participating classes are consented and screen positive - Retention of parents of children who screen positive at baseline at the first follow-up assessment is less than 80% but at least 70%. |
| **STOP:**  **We will consult the PSC regarding not progressing beyond the pilot if (any of the following):** | - Fewer than 18 pilot schools have been recruited - Less than 6% of Year 4 children in participating classes are consented and screen positive - Retention of parents of children who screen positive at baseline at the first follow-up assessment is less than 70%. - There have been serious adverse events that warrant closedown (based on consultation with the Programme Steering Committee). |

Supplement 3: Progression criteria assessed by the Programme Steering Committee prior to progressing to Phase 2

***We are expecting 40% of parents will complete the parent-report screening questionnaire, and 20% of these children will screen positive which overall means that we are expecting 8% of children in participating classes to screen positive (our target population)
